# Effectiveness of match-day priming strategies on physical and technical performance in female basketball players

**DOI:** 10.5114/biolsport.2026.159570

**Published:** 2026-07-06

**Authors:** Enrique Flórez-Gil, Nuno Mateus, Jaime Sampaio, Alejandro Vaquera, Eduardo Abade

**Affiliations:** 1 Faculty of Physical Activity and Sports Sciences, VALFIS Research Group, Institute of Biomedicine (IBIOMED), Universidad de León, León, Spain; 2 Research Center in Sports Sciences, Health Sciences and Human Development, CIDESD, Elite Re-search Community, Vila Real, Portugal; 3 Department of Sports Science, Exercise and Health, School of Life Sciences and Environment, University of Trás-os-Montes and Alto Douro, Vila Real, Portugal; 4 School of Sport & Exercise Science, University of Worcester, Worcester, United Kingdom; 5 FPF Academy, Portuguese Football Federation, Oeiras, Portugal

**Keywords:** Female athletes, Match-day readiness, Performance potentiation, Team sports, Post-activation performance enhancement

## Abstract

Priming refers to pre-competition training interventions designed to acutely enhance subsequent performance by optimizing neuromuscular, metabolic, and psychological readiness. The aim of this study was to compare the effects of three match-day priming strategies on physical and technical performance in female basketball players. Eighteen professional players (19.7 ± 2.1 years) from the Spanish third division completed a randomised crossover-controlled study involving four conditions: control (CON), high-load strength (STR; 80% 1RM), low-load power (POW; 40% 1RM), and plyometric with change-of-direction (PLY+COD). Performance was assessed in the morning (pre-test, 10:00 a.m.) and afternoon (post-test, 4:00 p.m.) using the countermovement jump (CMJ), drop jump (DJ), 5-0-5 change-of-direction speed (CODS) test, and a modified shooting-accuracy test. The POW condition resulted in significant improvements in CMJ, CODS, and shooting accuracy. In contrast, STR impaired CMJ, DJ height, CODS, and technical performance, while PLY+COD reduced jump performance and reactive strength. No meaningful changes occurred in CON. Complementary ANCOVA confirmed significant between-condition effects for CMJ, CODS, and shooting accuracy. These findings suggest that low-load, highvelocity resistance priming performed approximately 5 hours before competition can enhance neuromuscular readiness and shooting-accuracy in female basketball. Coaches should prioritise individualised, power-oriented priming routines aligned with players’ training backgrounds to optimise match-day performance.

## INTRODUCTION

Basketball is an intermittent team sport characterized by frequent bouts of high-intensity actions such as accelerations, decelerations, changes of direction, and jumps, interspersed with periods of lower activity and combined with complex technical-tactical skills [1, 2]. Given the multifactorial interaction of physical, technical-tactical, and contextual determinants of performance [3, 4], coaching staffs are required to design training and match-day strategies that optimise players’ readiness and performance.

Pre-match routines are central to this process, particularly when matches are scheduled for the afternoon or evening, as they influence both physical readiness and psychological activation [5, 6]. Within this context, exercise-based priming has emerged as an increasingly adopted approach in professional sport [7]. Unlike traditional warm-ups [8, 9], priming sessions are performed several hours before competition and aim to elicit delayed performance enhancements through short bouts of targeted exercise [10, 11]. These acute improvements are typically associated with mechanisms underpinning post-activation performance enhancement (PAPE), such as increased muscle temperature, elevated neural drive, improved excitation-contraction coupling, and hormonal responses including transient rises in testosterone [7, 10, 12, 13].

Despite these physiological advantages, research findings remain inconsistent. While some studies have demonstrated performance benefits following priming [14–16], others have found negligible or non-significant effects [17, 18]. Such discrepancies may stem from inter-individual variability in responsiveness, affected by training status, fibre-type composition, and recovery state, as well as from heterogeneous priming protocols [19].

Among the different modalities, resistance priming, typically involving low-volume, high-load compound movements, appears to be particularly effective in enhancing neuromuscular readiness and subsequent physical performance in team sports [7, 18, 20, 21]. Plyometric and power-based priming, characterized by explosive actions with moderate loads or bodyweight jumps, have also shown potential benefits for power output [7, 16]. Importantly, the success of any priming intervention is likely influenced by its alignment with the athlete’s habitual training load and individual physiological profile [22].

Beyond physical capacities, technical performance is a decisive component of basketball success, particularly shooting accuracy, which directly influences match outcomes [23–25]. The execution of technical skills in competitive situations is deeply influenced by neuromuscular preparation, coordination, and the level of fatigue of the players. This highlights the close connection between physical and technical domains [26].

Although a growing body of literature has explored priming effects in various sports, research in basketball remains limited, particularly among female players. Moreover, previous studies have largely concentrated on physical or physiological outcomes, with minimal attention given to sport-specific technical performance. Addressing this gap is particularly relevant in basketball, where optimal matchday strategies should support not only neuromuscular performance but also technical efficiency. Consequently, this study aimed to examine the effects of three distinct priming strategies, strength, power, and combined plyometric plus change-of-direction, on both the physical and technical performances of female basketball player.

## MATERIALS AND METHODS

### Subjects

Eighteen professional female basketball players (age: 19.7 ± 2.1 years; height: 176.1 ± 9.4 cm; body mass: 72.4 ± 12.0 kg) competing in the Spanish third division (Tier 3; McKay et al. [27]) voluntarily participated in the study. All players had a minimum of seven years of competitive experience and at least three years of structured strength training. Inclusion criteria required participation in a minimum of four weekly training sessions (≥ 90 min each) and one competitive match per week. During the pre-season period in which the study was conducted, overall training volume was relatively high, with an emphasis on technical–tactical content and physical preparation. During the competitive season, players typically completed 4–5 on-court training sessions per week (90 min each), complemented by 2–3 resistance-training sessions. Strength training was primarily oriented toward power and movement velocity, with most exercises performed at low to moderate loads (30–60% 1RM), while high-load resistance training (> 80% 1RM) was used less frequently. All participants were free from musculoskeletal, neurological, or orthopaedic injuries that could affect performance. The objectives and procedures of the study were explained in detail to all players and the coaching staff. Written informed consent was obtained from each participant, who were informed that participation was voluntary and that they could withdraw at any time without consequence. The study conformed to the ethical standards of the Declaration of Helsinki and received approval from the Ethics Committee of the University of León, Spain (reference number: ETICA-ULE-067-2024).

### Design and procedures

The study employed a randomised crossover-controlled design during the pre-season period, spanning four consecutive weeks, in which each player completed all four experimental conditions in a randomised order using simple randomisation: control (CON), strength priming (STR), power priming (POW), and plyometric plus change-of-direction priming (PLY+COD), performed on the same weekday each week to ensure consistency (Figure 1). All sessions were performed indoors on a regulation basketball court under consistent environmental conditions (temperature: ~21–22°C; relative humidity: ~45–55%). A familiarisation session took place one week before data collection to allow participants to become acquainted with all testing protocols and equipment.

**FIG. 1:**
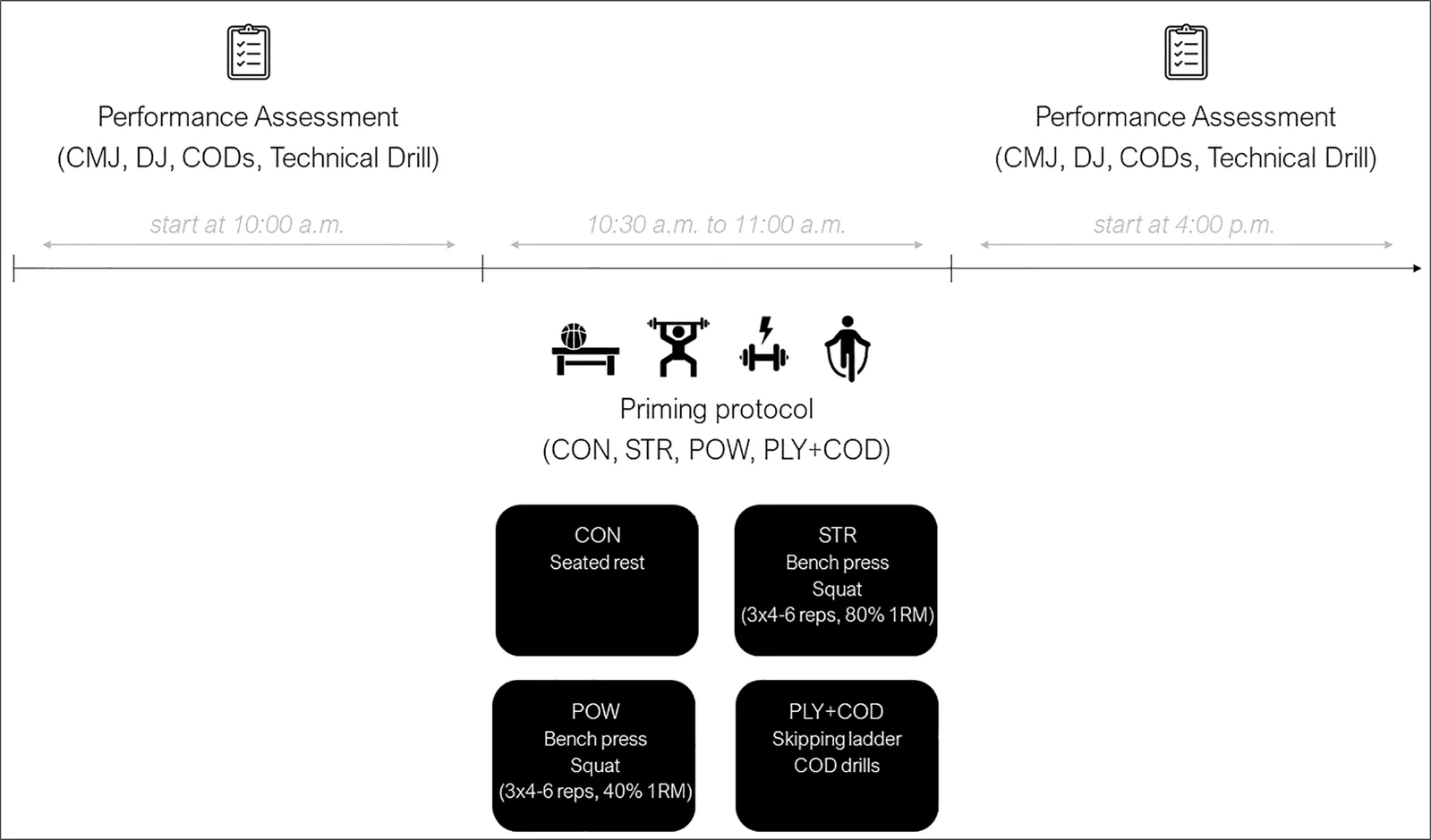
Study design illustrating the timeline of testing sessions and priming protocols. Players completed a morning performance assessment (CMJ, DJ, CODs, and technical drill) at 10:00 a.m., followed by one of four priming protocols between 10:30 and 11:00 a.m. (CON, STR, POW, or PLY+COD), and a second performance assessment at 4:00 p.m. Each condition was completed on separate days in a randomised order following a crossover design.

All priming protocols were designed to be time-matched, with a total duration of approximately 25–30 minutes per session, including warm-up, exercise execution, and rest intervals. In the CON condition, participants remained seated quietly for the duration of the priming period. The STR condition consisted of a high-load resistance training protocol comprising bench press and back squat exercises performed for three sets of six repetitions at 80% of one-repetition maximum (1RM). The POW condition followed an identical structure but with low-load exercises at 40% 1RM. In both strength and power sessions, all repetitions were executed with maximal concentric intent, with two-minute rest intervals between sets and exercises. The PLY+COD condition consisted of a combined plyometric and change-of-direction circuit adapted from Delextrat et al. [28]. Each repetition began with a two-foot skipping task performed through a coordination ladder (one foot contact per square), emphasising short ground contact times. Immediately after the ladder drill, players performed a basketball-specific change-of-direction circuit consisting of a short forward sprint (5 m), a lateral shuffle with two rapid 90° changes of direction, followed by a forward slalom around two cones (3 m appart), requiring two additional changes of direction. All movements were executed at maximal intended intensity. The circuit was repeated five times, with one-minute passive recovery between repetitions.

One-repetition maximums were previously determined during preseason testing. The priming sessions were performed approximately 5 hours before afternoon performance assessments to examine same-day post-activation performance enhancement effects.

### Performance Assessments

On each experimental day, players completed a battery of tests designed to evaluate both physical and technical performance, including the countermovement jump (CMJ), drop jump (DJ), 5-0-5 change-of-direction speed (CODS) test, and a modified medium- and long-range shooting test. Each player performed two trials per test, with 1 minute of rest between trials and two minutes between different assessments. The best performance in each test was retained for analysis. The full testing battery was administered twice on the same day: first in the morning (10:00 a.m.) to establish baseline performance, and again in the afternoon (4:00 p.m.) to assess performance following the respective priming intervention, which took place from 10:30 a.m. to 11 a.m. All performance assessments were performed in the same fixed order in both the morning and afternoon sessions to minimise the influence of accumulated fatigue on subsequent tests. This fixed sequence was selected to prioritise explosive power assessments before tasks involving higher metabolic and neuromuscular demands, in line with established testing battery recommendations [29]. Before each testing session, participants completed a standardised 10–12 min warm-up consisting of 5 min of low-intensity running, followed by dynamic mobility exercises for the lower and upper limbs, progressive submaximal jumps, and short accelerations. This warm-up was identical for all sessions and conditions and was designed to prepare participants for high-intensity efforts without inducing fatigue. This protocol enabled the examination of within-day performance variation and the delayed effects of each priming strategy.

The CMJ was used to assess lower-limb power. Players performed a rapid countermovement to approximately 90° of knee flexion before executing a maximal vertical jump while maintaining hands-on-hips positioning to minimise arm contribution [30]. The DJ was conducted unilaterally using the dominant leg, defined by self-report. Participants stepped off a 20 cm box, landed on the dominant leg, and immediately performed a maximal vertical jump, emphasising minimal ground contact time. Jump height and reactive strength index (RSI) were recorded using the MyJump 2.0 smartphone application, which has demonstrated high validity (r = 0.995, p ≤ 0.001) and reliability (ICC = 0.997, p ≤ 0.001) compared with force platforms [31].

The 5-0-5 CODS test was employed to assess change-of-direction ability [32]. Players started 0.5 m behind the start line, sprinted forward, executed a 180° turn around a cone, and accelerated back toward the finish line. Performance time was recorded using validated electronic photocells (Polifermo Light Radio, Microgate, Bolzano, Italy) positioned 3 m apart and 1 m above the floor.

The modified medium- and long-range shooting test evaluated shooting accuracy under physical exertion [33]. Players attempted 20 shots from ten designated positions on the court, alternating between 5 m and 6 m from the basket. After each shot, they sprinted to retrieve the ball and dribbled to the next shooting position. The test was completed within a 130-second time limit, and shooting accuracy was calculated based on successful attempts, adjusted for missed-shot penalties.

All assessments were conducted indoors under standardised environmental conditions, with consistent verbal encouragement provided to ensure maximal effort. The best recorded performance for each test was retained for subsequent analysis.

### Statistical Analysis

Firstly, a boxplot and a Shapiro-Wilk test were applied to all datasets to identify outliers and assess the normality of distributions. Cumming estimation plots were then generated to illustrate the magnitude and direction of performance changes between the morning (pre-test) and afternoon (post-test) assessments for each priming condition [34]. These graphical representations display individual values and the paired mean differences, accompanied by Cohen’s d effect sizes and corresponding 95% confidence intervals. Effect size thresholds were interpreted as trivial (< 0.2), small (0.2–0.59), moderate (0.6–1.19), large (1.2–2.0), and very large (> 2.0) [35]. Subsequently, a one-way analysis of covariance (ANCOVA) was performed to examine the influence of the different priming strategies on post-test performance outcomes. For each dependent variable, the corresponding pre-test score was included as a covariate, the priming strategy was entered as a fixed factor, and post-test performance served as the dependent measure. Effect sizes for the ANCOVA were expressed as partial eta squared (ηp^2^) and interpreted according to conventional thresholds: small (ηp^2^ ≥ 0.01), medium (ηp^2^ ≥ 0.06), and large (ηp^2^ ≥ 0.14) [36]. When significant main effects were detected, Bonferroni-adjusted post hoc comparisons were conducted to identify pairwise differences between priming conditions. All statistical analyses were performed using JASP software (Version 0.19.0; JASP Team, Amsterdam, Netherlands), with statistical significance set at *p* < 0.05.

## RESULTS

Descriptive and inferential statistics for all performance variables across the four priming strategies are presented in Table 1. Figure 2 illustrates the paired effect sizes (Cohen’s d) and 95% confidence intervals for performance changes between the morning (pre-test) and afternoon (post-test) assessments, providing a visual representation of the magnitude and direction of within-day variations.

**TABLE 1:** Descriptive and inferential analysis of players’ performance on the testing protocols according to the priming training strategies

Variables	Pre-test	Post-test	Cohen’s d [95% CI]	*p*
**CON**				
CMJ (cm)	28.62 (1.7)	28.75 (1.6)	0.08 [-0.06, 0.24]	0.272
DJ height (cm)	14.20 (2.6)	14.01 (2.3)	−0.07 [-0.41, 0.20]	0.599
DJ RSI (arb. u)	0.96 (0.2)	0.92 (0.1)	−0.33 [-0.67, -0.07]	0.049
CODs (sec)	2.28 (0.1)	2.28 (0.1)	0.05 [-0.21, 0.28]	0.634
Technical drill (counts)	27.67 (2.4)	27.83 (2.4)	0.07 [-0.29, 0.42]	0.594

**STR**				
CMJ (cm)	28.65 (1.7)	27.69 (1.5)	−0.59 [-0.91, -0.33]	< 0.001
DJ height (cm)	14.16 (1.9)	13.31 (2.0)	−0.43 [-0.88, -0.15]	0.010
DJ RSI (arb. u)	0.94 (0.1)	0.90 (0.1)	−0.28 [-0.73, 0.01]	0.118
CODs (sec)	2.27 (0.1)	2.30 (0.1)	0.43 [0.21, 0.67]	0.001
Technical drill (counts)	28.17 (2.5)	26.39 (2.0)	−0.79 [-1.31, -0.26]	0.005

**POW**				
CMJ (cm)	28.48 (1.5)	29.25 (1.7)	0.48 [0.34, 0.59]	0.001
DJ height (cm)	13.18 (2.1)	13.60 (2.4)	0.19 [-0.18, 0.66]	0.383
DJ RSI (arb. u)	0.91 (0.1)	0.91 (0.1)	−0.01 [-0.41, 0.50]	0.947
CODs (sec)	2.30 (0.1)	2.27 (0.1)	−0.38 [-0.64, -0.11]	0.005
Technical drill (counts)	27.41 (2.0)	28.53 (2.0)	0.55 [0.08, 1.02]	0.027

**PLY+COD**				
CMJ (cm)	28.65 (1.6)	27.52 (1.9)	−0.65 [-1.01, -0.26]	0.002
DJ height (cm)	13.78 (1.6)	13.18 (1.8)	−0.35 [-0.73, -0.04]	0.049
DJ RSI (arb. u)	0.95 (0.2)	0.89 (0.1)	−0.39 [-0.71, -0.14]	0.007
CODs (sec)	2.31 (0.1)	2.34 (0.1)	0.22 [-0.11, 0.48]	0.141
Technical drill (counts)	28.89 (2.2)	29.83 (2.6)	0.39 [0.0, 0.86]	0.060

Abbreviations: CON, absence of priming training; STR, priming via strength training; POW, priming via power training; PLY+COD, priming via plyometric and change-of-direction training; cm = centimeters; sec = seconds; arb. u = arbitrary units.

**FIG. 2:**
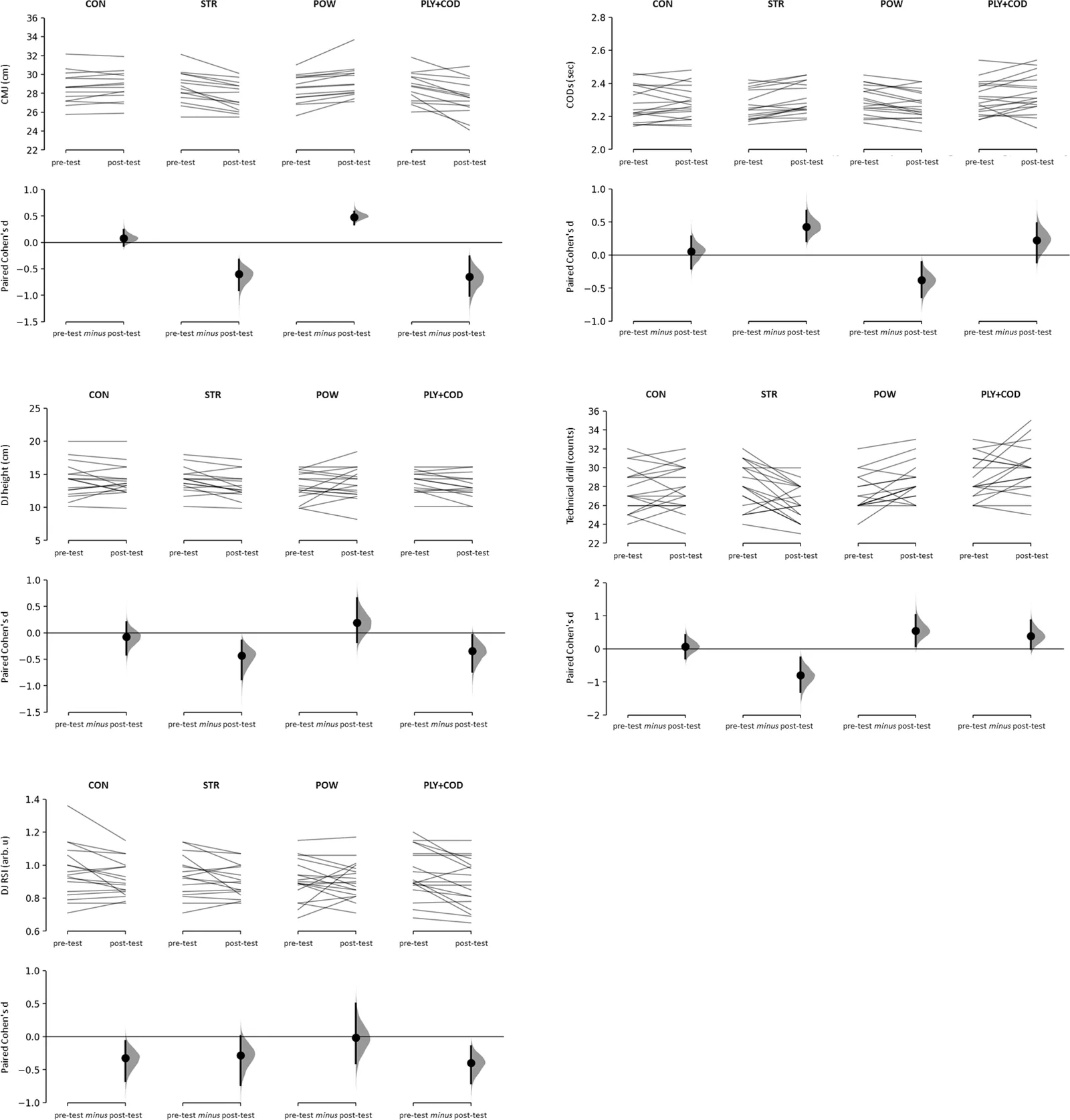
Cumming estimation plots illustrating the paired Cohen’s d effect sizes between the morning assessment (pre-test) and the afternoon assessment (post-test) across all priming conditions. The raw data are presented on the upper axes, with each paired observation connected by a line. The lower axes represent the paired Cohen’s d estimates with 95% confidence intervals, displayed as bootstrap sampling distributions. Each black dot represents the estimated effect size, with vertical error bars indicating the 95% confidence intervals. Legend: CON = control (seated rest); STR = strength priming (bench press and squat at 80% 1RM); POW = power priming (bench press and squat at 40% 1RM, explosive intent); PLY+COD = plyometric plus change-of-direction priming (skipping ladder and COD drills).

In the CON condition, no significant changes were observed in CMJ, DJ height, CODS, or technical drill performance (all *p* > 0.05). However, a small but statistically significant reduction was detected in DJ reactive strength index (RSI) from pre- to post-test (*p* = 0.049, d = -0.33, small effect).

In the STR condition, CMJ performance declined significantly after priming (*p* < 0.001, d = -0.59, small effect). Similarly, CODS performance deteriorated (*p* = 0.001, d = 0.43, small effect), and DJ height decreased (*p* = 0.010, d = -0.43, small effect). In contrast, technical drill performance decreased significantly (*p* = 0.005, d = -0.79, moderate effect), while DJ RSI showed no significant change (*p* = 0.118).

In the POW condition, CMJ performance improved significantly (*p* = 0.001, d = 0.48, small effect), accompanied by a reduction in CODS time, indicating enhanced change-of-direction ability (*p* = 0.005, d = -0.38, small effect). Technical drill performance also improved (*p* = 0.027, d = 0.55, small effect). No significant changes were found in DJ height (*p* = 0.383) or DJ RSI (*p* = 0.947).

In the PLY+COD condition, CMJ performance declined significantly (*p* = 0.002, d = -0.65, moderate effect), accompanied by a small but significant decrease in DJ height (*p* = 0.049, d = -0.35, small effect) and in DJ RSI (*p* = 0.007, d = -0.39, small effect). No significant differences were observed in CODS performance (*p* = 0.141) or technical drill accuracy (p = 0.060).

The ANCOVA results are summarised in Tables 2 and 3. Significant main effects of priming strategy were identified for CMJ performance (F = 18.48, *p* < 0.001, ηp^2^ = 0.52, large effect), CODS performance (F = 6.33, *p* < 0.001, ηp^2^ = 0.22, large effect), and technical drill execution (F = 9.81, *p* < 0.001, ηp^2^ = 0.31, large effect). No significant effects were observed for DJ height (F = 2.07, *p* = 0.114, ηp^2^ = 0.09) or DJ RSI (F = 0.99, *p* = 0.400, ηp^2^ = 0.05).

**TABLE 2:** Covariance (ANCOVA) analysis for the testing protocols variables across the different priming training strategies

Variables	*df*	*F*	*p*	*η*p^2^
CMJ (cm)	3	18.48	< 0.001	0.52
DJ height (cm)	3	2.07	0.114	0.09
DJ RSI (arb. u)	3	0.99	0.40	0.05
CODs (sec)	3	6.33	< 0.001	0.22
Technical drill (counts)	3	9.81	< 0.001	0.31

Abbreviations: cm = centimeters; sec = seconds; arb. u = arbitrary units; df = degrees of freedom; *η*p^2^ = partial Eta squared.

**TABLE 3:** Post hoc comparisons of the players’ performance on the testing protocols, considering the priming training strategies

Variables	Mean Difference [95% CI]	SE	t	*p*_tukey_
**CMJ (cm)**				
CON × STR	1.09 [0.31, 1.88]	0.30	3.69	0.003
CON × POW	-0.63 [-1.42, 0.16]	0.30	−2.12	0.160
CON × PLY+COD	1.26 [0.48, 2.05]	0.30	4.26	< 0.001
STR × POW	-1.72 [-2.51, -0.93]	0.30	−5.80	< 0.001
STR × PLY+COD	0.17 [-0.62, 0.96]	0.30	0.57	0.939
POW × PLY+COD	1.89 [1.10, 2.68]	0.30	6.38	< 0.001

**DJ height (cm)**				
CON × STR	0.67 [-0.58, 1.92]	0.47	1.42	0.494
CON × POW	−0.40 [-1.65, 0.85]	0.47	−0.85	0.831
CON × PLY+COD	0.50 [-0.78, 1.77]	0.48	1.03	0.732
STR × POW	−1.07 [-2.34, 0.20]	0.48	−2.24	0.126
STR × PLY+COD	−0.17 [-1.46, 1.12]	0.48	−0.36	0.985
POW × PLY+COD	0.90 [-0.38, 2.17]	0.48	1.86	0.257

**DJ RSI (arb. u)**				
CON × STR	-0.00 [-0.07, 0.07]	0.03	−0.14	0.999
CON × POW	-0.03 [-0.09, 0.04]	0.03	−0.97	0.768
CON × PLY+COD	0.02 [-0.05, 0.09]	0.03	0.73	0.884
STR × POW	-0.02 [-0.09, 0.05]	0.03	−0.81	0.847
STR × PLY+COD	0.02 [-0.05, 0.09]	0.03	0.87	0.823
POW × PLY+COD	0.04 [-0.02, 0.11]	0.03	1.72	0.320

**CODs (sec)**				
CON × STR	-0.03 [-0.08, 0.01]	0.02	−1.88	0.247
CON × POW	0.04 [-0.01, 0.08]	0.02	2.11	0.160
CON × PLY+COD	-0.02 [-0.07, 0.02]	0.02	−1.35	0.532
STR × POW	0.07 [0.02, 0.11]	0.02	3.94	0.001
STR × PLY+COD	0.01 [-0.04, 0.06]	0.02	0.53	0.952
POW × PLY+COD	-0.06 [-0.10, -0.01]	0.02	−3.48	0.005

**Technical drill (counts)**				
CON × STR	1.75 [0.16, 3.34]	0.60	2.91	0.025
CON × POW	−0.85 [-2.46, 0.75]	0.61	−1.40	0.504
CON × PLY+COD	−1.25 [-2.86, 0.36]	0.61	−2.05	0.180
STR × POW	−2.60 [-4.22, -0.99]	0.61	−4.25	< 0.001
STR × PLY+COD	−3.00 [-4.59, -1.41]	0.60	−4.98	< 0.001
POW × PLY+COD	−0.40 [-2.05, 1.25]	0.63	−0.64	0.918

Abbreviations: cm = centimeters; sec = seconds; arb. u = arbitrary units; SE = Standard Error; t = t-statistic.

Post hoc comparisons revealed significant differences in CMJ performance between CON and STR (*p* = 0.003, mean difference = 1.09), CON and PLY+COD (*p* < 0.001, mean difference = 1.26), STR and POW (*p* < 0.001, mean difference = -1.72), and POW and PLY+COD (*p* < 0.001, mean difference = 1.89). No significant differences were found between CON and POW or between STR and PLY+COD (both *p* > 0.05).

For CODS performance, significant differences were detected between STR and POW (*p* = 0.001, mean difference = 0.07) and between POW and PLY+COD (*p* = 0.005, mean difference = -0.06), with no other pairwise comparisons reaching significance (*p* > 0.05).

Regarding technical drill execution, significant differences were observed between CON and STR (*p* = 0.025, mean difference = 1.75), STR and POW (*p* < 0.001, mean difference = -2.60), and STR and PLY+COD (*p* < 0.001, mean difference = -3.00). No other pairwise comparisons showed statistically significant differences (*p* > 0.05).

## DISCUSSION

This study investigated the effects of different match-day priming strategies on the physical and technical performance of female basketball players. The findings revealed that both STR (i.e., high-load strength) and PLY+COD (i.e., plyometric plus change-of-direction) routines impaired afternoon performance, whereas the POW (i.e., low-load power) strategy effectively enhanced jumping, running, and technical abilities 5 hours after implementation. Specifically, the use of high-load resistance training at 80%1RM and ladder drills combined with high-intensity change-of-direction activities led to performance decrements, while the low-load (40%1RM) protocol improved countermovement jump height, change-of-direction speed, and shooting accuracy.

Previous research has shown that resistance-based priming can elicit delayed neuromuscular potentiation when performed within a 6-hour window before competition [7]. Such effects, often described as PAPE, may be achieved using either high-load (≥ 85%1RM) or low-load (30–40%1RM) ballistic exercises [37]. However, the magnitude and direction of the response appear to depend on several factors, including exercise type, training volume, recovery interval, individual strength level, and prior training exposure [38]. Consequently, inter-individual variability is common, and players may respond differently to the same priming stimulus, a pattern observed in the present investigation (see Figure 2). These findings support the notion that priming routines should be individualised not only in terms of load prescription but also according to an athlete’s specific training history and preferences [10].

Although high-load resistance priming has been reported to enhance performance in some contexts, the effectiveness of such protocols appears to depend strongly on players’ strength levels and tolerance to heavy loading. For instance, Cook et al. [18] observed that rugby union players, who possess high absolute strength, improved performance 6 hours after performing bench press and squat exercises at 50, 80, 90 and 100% of 3RM. Similarly, González-García et al. [39] reported positive relationships between strength level and improvements in jump height and concentric velocity following highload squats. However, when similar loads were applied to female rugby players, Loturco et al. [16] found increased perceived exertion and reduced gains in speed and power-related outcomes after priming sessions conducted 6–24 hours before activity. Although rate of perceived exertion was not assessed in the present study, the observed impairments in both physical and technical performance 5 hours after the high-load protocol suggest that fatigue likely outweighed any potentiation effects.

In contrast, the low-load power condition (POW), consisting of bench press and squat exercises at 40%1RM performed with maximal concentric intent, produced consistent performance improvements. These findings align with previous reports demonstrating that low- to moderate-load priming (30–65%1RM), particularly when performed with ballistic intent, can enhance neuromuscular performance [7]. Although the underlying mechanisms remain uncertain, acute reductions in joint stiffness, increased neural activation, and transient elevations in testosterone have been proposed as potential contributors [7, 10]. It is also plausible that the participants’ prior experience, characterised by several years of power- and velocityoriented resistance training, optimised their responsiveness to lowload, high-velocity movements. Conversely, their limited exposure to heavy-load resistance training may have increased fatigue susceptibility, attenuating potential potentiation effects. Indeed, players with less experience in high-load training appear more likely to experience fatigue than performance enhancement following such priming routines [38].

Beyond the physical domain, match-day priming may also influence technical execution through its effects on neuromuscular readiness, coordination, and fatigue management [40]. To the best of our knowledge, this is the first study to examine the effects of different match-day priming strategies on technical performance in basketball. The improvements in shooting accuracy observed under the POW condition occurred alongside improvements in physical performance, suggesting a more favourable neuromuscular state at the time of testing. This increased physical readiness may have facilitated greater movement control, temporal accuracy, and shooting mechanics, which are known to be sensitive to fatigue and neuromuscular alterations. In contrast, the decrease in performance observed following STR and PLY+COD preparation may reflect residual fatigue that negatively affected both physical performance and technical execution. Taken together, these findings support the idea that low-load, high-speed preparation can improve technical readiness by optimising neuromuscular function without inducing excessive fatigue, providing practical assurance to coaches seeking to implement match-day strategies that optimise both physical and technical readiness.

Several limitations should be considered when interpreting these results. The study was conducted within a single basketball club, and findings may therefore reflect the specific training background of this cohort. Consequently, the generalisability of these outcomes may be limited to players with similar profiles and competition levels. Future research should aim to replicate and extend these findings across larger and more heterogeneous samples, and to integrate physiological and perceptual markers such as muscle temperature, hormonal responses (e.g., testosterone), oxygen uptake, and heart rate [41]. In addition, internal load and acute neuromuscular responses were not assessed; measures such as RPE or session-RPE, as well as immediate post-priming CMJ assessments, could have provided valuable information regarding fatigue and activation mechanisms. Menstrual cycle phase was not controlled, which may have contributed to additional variability in performance responses. Although a familiarisation session was conducted, and the players are accustomed to shooting task, the test-retest reliability of the modified shooting-accuracy test was not formally established, and internal fatigue during the task was not directly monitored. Therefore, improvements in shooting performance may have been partially influenced by repeated exposure to the test or by day-to-day motivational fluctuations, although the randomised, counterbalanced crossover design aimed to minimise systematic learning or order effects. Such analyses would provide deeper insight into the mechanisms mediating the relationship between priming load, fatigue, and subsequent performance.

## CONCLUSIONS

Low-load, high-velocity resistance priming, performed approximately 5 hours before competition, proved to be an effective strategy to enhance both physical performance and shooting accuracy in female basketball players. In contrast, high-load and combined plyometric plus change-of-direction routines resulted in transient impairments, emphasising the importance of selecting match-day strategies that minimise fatigue while promoting neuromuscular readiness. Accordingly, coaches and practitioners are encouraged to prioritise individualised priming routines that align with each athlete’s physiological characteristics and training background, thereby facilitating optimal performance without detriment to technical execution.

## References

[1] Wang C, Ye M. Individualizing Basketball-Specific Interval Training Using Anaerobic Speed Reserve: Effects on Physiological and Hormonal Adaptations. Int J Sports Physiol Perform. 2024; 19(4):365–74. 10.1123/ijspp.2023-0379 38237576

[2] Puente C, Abián-Vicén J, Areces F, López R, Coso J Del. Physical and Physiological Demands of Experienced Male Basketball Players During a Competitive Game. J Strength Cond Res. 2017; 31(4):956–62. 10.1519/JSC.0000000000001577 27467516

[3] Mateus N, Gonçalves B, Abade E, Liu H, Torres-Ronda L, Leite N, Sampaio J. Game-to-game variability of technical and physical performance in NBA players. Int J Perform Anal Sport. 2015; 15(3):764–76.

[4] Stojanović E, Stojiljković N, Scanlan AT, Dalbo VJ, Berkelmans DM, Milanović Z. The Activity Demands and Physiological Responses Encountered During Basketball Match-Play: A Systematic Review. Sports Medicine. 2018; 48(1):111–35. 10.1007/s40279-017-0794-z 29039018

[5] Modric T, Carling C, Lago-Peñas C, Versic Š, Morgans R, Sekulic D. To train or not to train (on match day)? Influence of a priming session on match performance in competitive elite-level soccer. J Sports Sci. 2023; 41(18):1726–33. 10.1080/02640414.2023.2296741 38115752

[6] Donghi F, Rampinini E, Bosio A, Fanchini M, Carlomagno D, Maffiuletti NA. Morning Priming Exercise Strategy to Enhance Afternoon Performance in Young Elite Soccer Players. Int J Sports Physiol Perform. 2021; 16(3):407–14. 10.1123/ijspp.2020-0094 33401241

[7] Harrison PW, James LP, McGuigan MR, Jenkins DG, Kelly VG. Resistance Priming to Enhance Neuromuscular Performance in Sport: Evidence, Potential Mechanisms and Directions for Future Research. Sports Medicine. 2019; 49(10):1499–514. 10.1007/s40279-019-01136-3 31203499

[8] Flórez-Gil E, Mateus N, Sampaio J, Abade E. Examining the impact of different re-warm-up strategies on non-starter basketball players’ physical performance. J Sports Sci. 2025; 43(6):536–43. 10.1080/02640414.2025.2467564 39960231

[9] Flórez-Gil E, Boullosa D, Calleja-González J, Vaquera A. A Cross-National Survey of Basketball Strength and Conditioning Coaches’ Warm-Up Practices. Int J Sports Physiol Perform. 2025; 8(11):1501–8. 10.1123/ijspp.2025-0045 40921425

[10] Mason B, McKune A, Pumpa K, Ball N. The Use of Acute Exercise Interventions as Game Day Priming Strategies to Improve Physical Performance and Athlete Readiness in Team-Sport Athletes: A Systematic Review. Sports Medicine. 2020; 50(11):1943–62. 10.1007/s40279-020-01329-1 32779102

[11] Woolstenhulme MT, Kerbs Bailey B, Allsen PE. Vertical jump, anaerobic power, and shooting accuracy are not altered 6 hours after strength training in collegiate women basketball players. J Strength Cond Res. 2004; 18(3):422–5. 10.1519/13463.1 15320641

[12] Finlay MJ, Bridge CA, Greig M, Page RM. Upper-Body Post-activation Performance Enhancement for Athletic Performance: A Systematic Review with Meta-analysis and Recommendations for Future Research. Sports Medicine. 2022; 52(4):847–71. 10.1007/s40279-021-01598-4 PMC8938350 34826109

[13] Biel P, Ewertowska P, Stastny P, Krzysztofik M. Effects of Complex Training on Jumping and Change of Direction Performance, and Post-Activation Performance Enhancement Response in Basketball Players. Sports. 2023; 11(9). 10.3390/sports11090181 PMC10534482 37755858

[14] Marrier B, Durguerian A, Robineau J, Chennaoui M, Sauvet F, Servonnet A, Piscione J, Mathieu B, Peeters A, Lacome M, Morin JB, Le Meur Y. Preconditioning strategy in rugby-7s players: Beneficial or detrimental? Int J Sports Physiol Perform. 2019; 14(7):918–26. 10.1123/ijspp.2018-0505 30569798

[15] Pino-Mulero V, Soriano MA, Giuliano F, González-García J. Effects of a priming session with heavy sled pushes on neuromuscular performance and perceived recovery in soccer players: a crossover design study during competitive microcycles. Biol Sport. 2025; 42(1):59–66. 10.5114/biolsport.2025.139082 PMC11694209 39758171

[16] Loturco I, Zmijewski P, Mercer VP, Ramos MS, Betelli MT, Arenhart I, Moura TBMA, Pereira LA. Effects of light-load vs. heavy-load jump squats as priming activities in Olympic female rugby sevens players. Biol Sport. 2025; 42(2):85–94. 10.5114/biolsport.2025.142645 PMC11963119 40182726

[17] Russell M, Williams N, Kilduff LP. Advanced Strength and Conditioning. London, UK: Rotuledge; 2017.

[18] Cook CJ, Kilduff LP, Crewther BT, Beaven M, West DJ. Morning based strength training improves afternoon physical performance in rugby union players. J Sci Med Sport. 2014; 17(3):317–21. 10.1016/j.jsams.2013.04.016 23707139

[19] Harrison PW, James LP, McGuigan MR, Jenkins DG, Kelly VG. Prevalence and application of priming exercise in high performance sport. J Sci Med Sport. 2020; 23(3):297–303. 10.1016/j.jsams.2019.09.010 31594712

[20] Schelling X, Torres-Ronda L. An Integrative Approach to Strength and Neuromuscular Power Training for Basketball. Strength Cond J. 2016; 38(3):72–80.

[21] Ibáñez SJ, Piñar MI, García D, Mancha-Triguero D. Physical Fitness as a Predictor of Performance during Competition in Professional Women’s Basketball Players. Int J Environ Res Public Health. 2023; 20(2):988. 10.3390/ijerph20020988 PMC9859287 36673748

[22] Holmberg PM, Russell S, O’Brien KA, James LP, Kelly VG. Exploring strength and conditioning practitioners’ perceptions about using priming exercise as a pre-competition strategy to improve performance. Int J Sports Sci Coach. 2024; 19(4):1598–611.

[23] Zhang M, Miao X, Rupčić T, Sansone P, Vencúrik T, Li F. Determining the Relationship between Physical Capacities, Metabolic Capacities, and Dynamic Three-Point Shooting Accuracy in Professional Female Basketball Players. Applied Sciences (Switzerland). 2023; 13(15).

[24] Espasa-Labrador J, Martínez-Rubio C, García F, Fort-Vanmeergaehe A, Guarch J, Calleja-González J. Exploring the Relationship Between Game Performance and Physical Demands in Youth Male Basketball Players. J Funct Morphol Kinesiol. 2025; 10(3). 10.3390/jfmk10030293 PMC12371998 40843824

[25] Pojskic H, Sisic N, Separovic V, Sekulic D. Association Between Conditioning Capacities and Shooting Performance in Professional Basketball Players: An Analysis of Stationary and Dynamic Shooting Skills. J Strength Cond Res. 2018; 32(7):1981–92. 10.1519/JSC.0000000000002100 29939949

[26] Martinho D V., Clemente FM, Ángel-Gomez M, Rebelo A, Field A, Santos CC, Gouveia ÉR, Afonso J, Sarmento H. Physical, Physiological, Technical and Tactical Responses According to the Playing Position in Male Basketball: A Systematic Scoping Review. J Hum Kinet. 2025; 96:5–35. 10.5114/jhk/203326 PMC12121883 40453900

[27] McKay AKA, Stellingwerff T, Smith ES, Martin DT, Mujika I, Goosey-Tolfrey VL, Sheppard J, Burke LM. Defining Training and Performance Caliber: A Participant Classification Framework. Int J Sports Physiol Perform. 2022; 17(2):317–31. 10.1123/ijspp.2021-0451 34965513

[28] Delextrat A, Grosgeorge B, Bieuzen F. Determinants of performance in a new test of planned agility for young elite basketball players. Int J Sports Physiol Perform. 2015; 10(2):160–5. 10.1123/ijspp.2014-0097 24956606

[29] Turner A, Walker S, Stembridge M, Coneyworth P, Reed G, Birdsey L, Barter P, Moody J. A Testing Battery for the Assessment of Fitness in Soccer Players. Strength Cond J. 2011; 33(5):29–39.

[30] Van Hooren B, Zolotarjova J. The Difference Between Countermovement and Squat Jump Performances: A Review of Underlying Mechanisms With Practical Applications. J Strength Cond Res. 2017; 31(7):2011–20. 10.1519/JSC.0000000000001913 28640774

[31] Balsalobre-Fernández C, Glaister M, Lockey RA. The validity and reliability of an iPhone app for measuring vertical jump performance. J Sports Sci. 2015; 33(15):1574–9. 10.1080/02640414.2014.996184 25555023

[32] Haff Greg, Triplett NTravis. Essentials of strength training and conditioning. 4^th^ ed. Human Kinetics; 2015.

[33] Matulaitis K, Skarbalius A, Abrantes C, Gonçalves B, Sampaio J. Fitness, technical, and kinanthropometrical profile of youth Lithuanian basketball players aged 7–17 years old. Front Psychol. 2019; 10. 10.3389/fpsyg.2019.01677 PMC6646714 31379689

[34] Ho J, Tumkaya T, Aryal S, Choi H, Claridge-Chang A. Moving beyond P values: data analysis with estimation graphics. Nat Methods. 2019; 16(7):565–6. 10.1038/s41592-019-0470-3 31217592

[35] Hopkins WG, Marshall SW, Batterham AM, Hanin J. Progressive statistics for studies in sports medicine and exercise science. Med Sci Sports Exerc. 2009; 41(1):3–12. 10.1249/MSS.0b013e31818cb278 19092709

[36] Cohen J. Statistical power analysis for the behavioral sciences. Cambridge: Academic Press; 2013.

[37] Xu K, Blazevich AJ, Boullosa D, Ramirez-Campillo R, Yin MY, Zhong YM, Tian YH, Finlay M, Byrne PJ, Cuenca-Fernández F, Wang R. Optimizing Post-activation Performance Enhancement in Athletic Tasks: A Systematic Review with Meta-analysis for Prescription Variables and Research Methods. Sports Medicine. 2025; 55(4):977–1008. 10.1007/s40279-024-02170-6 39853660

[38] Blazevich AJ, Babault N. Post-activation Potentiation Versus Post-activation Performance Enhancement in Humans: Historical Perspective, Underlying Mechanisms, and Current Issues. Front Physiol. 2019; 10:1359. 10.3389/fphys.2019.01359 PMC6838751 31736781

[39] González-García J, Latella C, Aguilar-Navarro M, Romero-Moraleda B. Effects of Resistance Priming Exercise on Within-day Jumping Performance and its Relationship with Strength Level. Int J Sports Med. 2023; 44(1):38–47. 10.1055/a-1898-4888 35820447

[40] Turner A, Comfort P. Advanced strength and conditioning: An Evidence-based Approach. London, UK: Routledge; 2022.

[41] Nutt F, Hills SP, Russell M, Waldron M, Scott P, Norris J, Cook CJ, Mason B, Ball N, Kilduff LP. Morning resistance exercise and cricket-specific repeated sprinting each improve indices of afternoon physical and cognitive performance in professional male cricketers. J Sci Med Sport. 2022; 25(2):162–6. 10.1016/j.jsams.2021.08.017 34535402

